# Intestinal Concretion

**Published:** 1845-03

**Authors:** 


*Intestinal concretion.*—At a meeting of the Sheffield Medical Society, Mr. Reedal exhibited a portion of the colon, and also a concretion of feculent matter, taken from the body of a man who died after a few days' illness, suffering with symptoms of ileus. Many years ago, he had taken, by mistake, an ounce of carbonate of potash, which made him very ill for some time, and shortly afterwards he perceived a tumour in the left iliac region, which was treated for hernia. On one occasion, having suffered from constipation, he was removed some distance in a cart, by the jolting of which it appeared as if something had been removed, as the bowels were freely opened. On examination after death, the ascending colon was found to be very much distended and thickened, and when opened, presented the appearance of inflammation with ulceration of the mucous lining. In the cæcum was found the concretion, which weighed four ounces, and measured six inches in circumference. On a section being made, it was found to be composed of feculent matter, in concentric layers of a light brown colour; externally it was dark, nearly approaching to black.—*London Medical Times..*

